# Complete Chloroplast Genome Sequence of Omani Lime (*Citrus aurantiifolia*) and Comparative Analysis within the Rosids

**DOI:** 10.1371/journal.pone.0113049

**Published:** 2014-11-14

**Authors:** Huei-Jiun Su, Saskia A. Hogenhout, Abdullah M. Al-Sadi, Chih-Horng Kuo

**Affiliations:** 1 Institute of Ecology and Evolutionary Biology, National Taiwan University, Taipei, Taiwan; 2 Department of Cell and Developmental Biology, John Innes Centre, Norwich, United Kingdom; 3 Department of Crop Sciences, Sultan Qaboos University, Al Khoud, Oman; 4 Institute of Plant and Microbial Biology, Academia Sinica, Taipei, Taiwan; 5 Molecular and Biological Agricultural Sciences Program, Taiwan International Graduate Program, National Chung Hsing University and Academia Sinica, Taipei, Taiwan; 6 Biotechnology Center, National Chung Hsing University, Taichung, Taiwan; Nanjing Forestry University, China

## Abstract

The genus *Citrus* contains many economically important fruits that are grown worldwide for their high nutritional and medicinal value. Due to frequent hybridizations among species and cultivars, the exact number of natural species and the taxonomic relationships within this genus are unclear. To compare the differences between the *Citrus* chloroplast genomes and to develop useful genetic markers, we used a reference-assisted approach to assemble the complete chloroplast genome of Omani lime (*C*. *aurantiifolia*). The complete *C*. *aurantiifolia* chloroplast genome is 159,893 bp in length; the organization and gene content are similar to most of the rosids lineages characterized to date. Through comparison with the sweet orange (*C. sinensis*) chloroplast genome, we identified three intergenic regions and 94 simple sequence repeats (SSRs) that are potentially informative markers with resolution for interspecific relationships. These markers can be utilized to better understand the origin of cultivated *Citrus*. A comparison among 72 species belonging to 10 families of representative rosids lineages also provides new insights into their chloroplast genome evolution.

## Introduction

*Citrus* is in the family of Rutaceae, which is one of the largest families in order Sapindales. Flowers and leaves of *Citrus* are usually strong scented, the extracts from which contain many useful flavonoids and other compounds that are effective insecticides, fungicides and medicinal agents [1]–[3]. *Citrus* is of great economic importance and contains many fruit crops such as oranges, grapefruit, lemons, limes, and tangerines. However, due to a long cultivation history, wide dispersion, somatic bud mutation, and sexual compatibility among *Citrus* species and related genera, the taxonomy of *Citrus* remains controversial [4], [5] and the origination of many *Citrus* species and hybrids is still unresolved [6], [7].

The chloroplast (cp) genome sequence contains useful information in plant systematics because of its maternal inheritance in most angiosperms [8], [9] and its highly conserved structures for developing promising genetic markers. The only complete cp genome available in *Citrus* is sweet orange (*Citrus sinensis*) [10], which has provided valuable information to the position of Sapindales in rosids. Although a genome sequencing project is in progress for *C. clementine*, its complete chloroplast genome sequence is not available yet. To identify the cp genome regions that are polymorphic and may be used as molecular markers for resolving the evolutionary relationships among *Citrus* species, a second cp genome within the genus is necessary for comparative analysis. For this purpose, the major aim of this study is to determine the complete cp genome sequence of *C. aurantiifolia*.

*C. aurantiifolia*, which is commonly known as Key lime, Mexican lime, Omani lime, Indian lime, or acid lime, is native to Southeast Asia and widely cultivated in tropics and subtropics. Oman is known to be a transit country for lime, from which lime spread to Africa and the New World [11]. In Oman, Omani lime is considered the fourth most important fruit crop in terms of cultivated area and production. The products of Omani lime can be used for beverage, food additives and cosmetic industries [12]. Omani lime is sensitive to several biotic agents, the most serious of which is ‘*Candidatus* Phytoplasma aurantifolia’, the cause of witches’ broom disease of lime (WBDL). Recent studies on WBDL focused on effect of genetic diversity of Omani limes on the disease [13], transcriptome and proteomic analysis of lime response to infection by phytoplasma [14]–[16] and effect of phytoplasma on seed germination, growth and metabolite content in lime [17], [18].

Here, we present the complete chloroplast genome sequence of Omani lime (*C. aurantiifolia*). To identify loci of potential utility for the molecular identification and phylogenetic analyses of *Citrus* cultivars and species, we compared the intergenic regions and SSRs in the cp genomes of *C*. *aurantiifolia* and *C. sinensis*. Furthermore, we performed phylogenetic analyses to infer the history of gene losses in the cp genome evolution among representative rosids lineages.

## Materials and Methods

### Sample Preparation and Sequencing

The Omani lime leaves were collected from a 5-year-old lime tree at a private farm located in the Omani territory of Madha (GPS coordinates: 25.276318, 56.318909). This farm is owned by one of the co-authors of this work, Dr. Abdullah M. Al-Sadi, whom should be contacted for future permissions. This study does not involve endangered or protected species and does not require specific permission from regulatory authority concerned with protection of wildlife. The sample was stored in a cool box and transported to the Plant Pathology Research Laboratory at Sultan Qaboos University (Al Khoud, Oman) for DNA extraction following a protocol of Maixner et al. [19]. The leaves were washed with clear water before the isolation procedure. 1 g of leaves were used and crushed in 3 ml CTAB extraction buffer (2% CTAB, 1.4 M NaCl, 500 mM EDTA pH8, 1 M Tris-HCl pH8 and 0.2% beta-mercaptol). 1.5 ml of the leave extract was transferred to a 2 ml tube and incubated in a water-bath at 65°C for 15 min. The tube was turned up and down twice during incubation, centrifuged at 960 g for 5 min, and the supernatant was subsequently transferred to a clean eppendorf tube. An equal volume of chloroform-isoamyl alcohol mix (24∶1) was added and the tube was centrifuged at 21000 g for 20 min. The supernatant was transferred to a new tube and then 0.6 volume of isopropanol was added to the supernatant and incubated at −20°C for 30 min. The DNA pellet was collected by centrifugation at 21000 g for 20 min and then washed with 1 ml of 70% ethanol. The final DNA was resuspended in 100 µl TE (Tris 10 mM, EDTA 1 mM pH8) and was stored at −80°C until used.

The library construction and sequencing were done at the Genome Analysis Centre (Norwich, UK). The Illumina TruSeq DNA Sample Preparation v2 Kit was used to prepare an indexed library. The DNA sample was sheared to a fragment size of 500–600 bp using a sonicator, followed by end-repair and the addition of a single A base for binding of the indexed adapter. The appropriate sized library (500 bp) was selected by gel electrophoresis, followed by PCR enrichment. The 251 bp paired-end sequencing run was performed on an Illumina MiSeq instrument using the SBS chemistry and Illumina software MCS v2.3.0.3 and RTA v1.18.42. The raw reads were deposited at the NCBI Sequence Read Archive under the accession number SRR1611615.

### Genome Assembly and Analyses

The procedures for genome assembly and annotation were based on our previous studies of cp genomes [20], [21]. In addition to the standard *de novo* assembly approach by using Velvet v1.2.10 [22] with the k-mer size set to 243, a reference-based approach for assembly as described below was used in parallel. All of the raw reads were initially mapped onto the published cp genome of *C. sinensis* [10] using BWA v0.6.2 [23]. The sequence variations were identified with SAMtools v0.1.19 [24] and visually inspected using IGV v2.3.25 [25]. The variants were corrected with the raw reads and the regions without sufficient coverage were converted into gaps. This corrected sequence was then used as the new draft reference for the next iteration of verification. Gaps were filled using the reads overhang at margins and the process was repeated until the reference was fully supported by all mapped raw reads. The final assembly, which was supported by our *de novo* and reference-based approaches, resulted in an average of 1,441-fold coverage of paired-end reads with a mapping quality of 60 and the region with the lowest coverage is 506-fold.

The preliminary annotations of the *C*. *aurantiifolia* cp genome were performed online using the automatic annotator DOGMA [26] and verified using BLASTN [27], [28] searches (e-value cutoff = 1e-10) against other land plant cp genomes. Each annotated gene was manually compared with *C. sinensis* cp genome for start and stop codons or intron junctions to ensure accurate annotation. The codon usage was analyzed by using the seqinr R-cran package [29]. A circular map of genome was produced using OGDRAW [30].

To identify the differences between *C*. *aurantiifolia* and *C. sinensis,* the two sequences were aligned using Mauve v2.3.1 [31] and the result was analyzed using custom Perl scripts. Intergenic gene regions were parsed out from the two *Citrus* cp genomes and aligned using MUSCLE v3.8.31 [32] with the default settings. The pairwise distances were calculated using the DNADIST program in the PHYLIP package v3.695 [33].

The positions and types of simple sequence repeats (SSRs) in the two *Citrus* cp genomes were detected using MISA (http://pgrc.ipk-gatersleben.de/misa/). The minimum number of repeats were set to 10, 5, 4, 3, 3, and 3 for mono-, di-, tri-, tetra-, penta-, and hexanucleotides, respectively. For long repeats, the program REPuter [34] was used to identify the number and location of direct and inverted (i.e., palindromic) repeats. A minimum repeat size of 30 bp and sequence identity greater than 90% setting were used according to the study of *C. sinensis* cp genome [10]. The redundant or overlapping repeats were identified and filtered manually.

### Phylogenetic Inference

Phylogenetic analysis of the representative rosids lineages with complete cp genomes available was performed using PhyML v20120412 [35] with the GTR+I+G model. A total of 72 rosids species were chosen as the ingroups and *Vitis venifera* was included as the outgroup, the accession numbers were provided in Table S1. The protein-coding and rRNA genes were parsed from the selected cp genomes and clustered into ortholog groups using OrthoMCL [36]. The presence/absence of orthologous genes in each genome was examined and further verified using TBLASTN [27], [28] searches (e-value cutoff = 1e-10). The nucleotide sequences of the conserved genes were aligned individually by using MUSCLE with the default settings. The concatenated alignment was used to infer a maximum likelihood phylogeny as described above. The bootstrap supports were estimated from 1,000 resampled alignments generated by the SEQBOOT program in the PHYLIP package.

### Investigations of *orf56* and *ycf68*

To investigate the presence/absence of *orf56* and *ycf68* in the selected cp genomes, the gene sequences from *C. aurantiifolia* was used as the queries to perform BLASTN [27], [28] searches (e-value cutoff = 1e-10). The significant hits were examined to investigate the presence of intact open reading frames (ORFs). Phylogenetic analysis of the cp *orf56* genes and the homologous mitochondrial sequences was performed as described above. The final alignment contains 190 aligned nucleotide sites and a total of 70 sequences, including two sequences of *Amborella* as the outgroup.

## Results and Discussion

### General Features of the Omani Lime Chloroplast Genome

**Figure 1:**
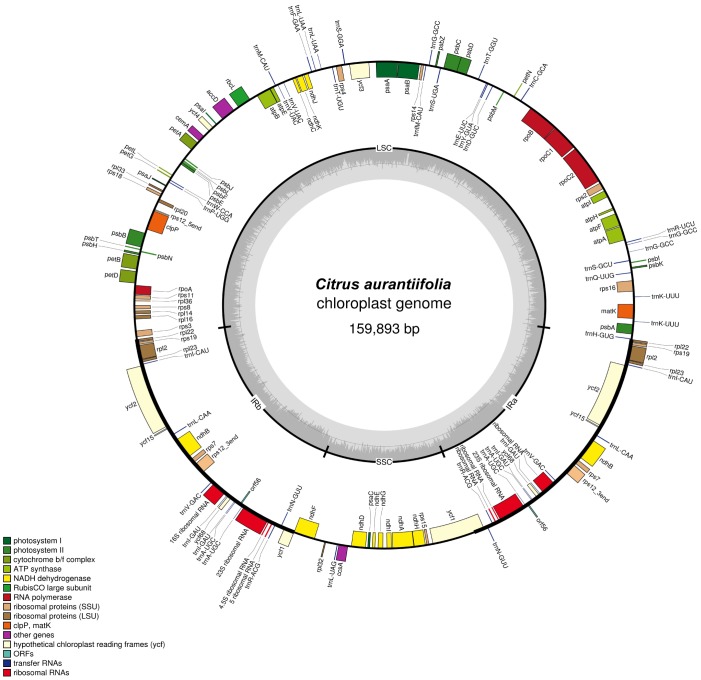
Chloroplast genome map of *Citrus aurantiifolia*. Gene drawn inside the circle are transcribed clockwise, whereas those outside are counterclockwise. The within-genome GC content variation is indicated in the middle circles.

The complete cp genome of *C*. *aurantiifolia* (Christm.) Swingle (GenBank accession number KJ865401.1) is 159,893 bp in length, including a large single copy (LSC) region of 87,148 bp, a small single copy (SSC) region of 18,763 bp, and a pair of inverted repeats (IRa and IRb) of 26,991 bp each (Figure 1 and Table 1). A total of 137 different genes, including 93 protein-coding genes, 30 tRNA genes, and four rRNA genes, were annotated (Table S2). Among these, 12 protein-coding genes and 7 tRNA genes are duplicated in the IR regions. Most of the protein-coding genes are composed of a single exon, while 14 contain one intron and three contain two introns. The gene *rps12* was predicted to undergo trans-splicing, with the 5′ exon located in the LSC region and the other two exons located in the IR regions.

**Table 1:** Summary of the *Citrus* chloroplast genome characteristics.

Attribute	*C. aurantiifolia* (KJ865401)	*C. sinensis* (NC_008334)
Size (bp)	159,893	160,129
overall GC content (%)	38.4	38.5
LSC size in bp (% total)	87,148 (54.5%)	87,744 (54.8%)
SSC size in bp (% total)	18,763 (11.7%)	18,393 (11.5%)
IR size in bp (% total)^a^	26,991 (16.9%)	26,996 (16.9%)
Protein-coding regions size in bp (% total)	81,468 (51.0%)	79,773 (49.8%)
rRNA and tRNA size in bp (% total)	11,850 (7.5%)	11,850 (7.4%)
Introns size in bp (% total)	17,129 (10.7%)	18,252 (11.4%)
Intergenic spacer size in bp (% total)	49,446 (30.9%)	50,254 (31.4%)
Number of different genes	115	113^b^
Number of different protein-coding genes	81	79^b^
Number of different rRNA genes	4	4
Number of different tRNA genes	30	30
Number of different genes duplicated by IR	22	20
Number of different genes with introns	17	17

a Each cp genome contains two copies of inverted repeats (IRs).

b According to the original annotation, not including *orf56*.

The protein-coding regions contain a total of 27,159 codons (Table S3). Isoleucine and cysteine are the most and least frequent amino acids and have 2,892 (10.7%) and 359 (1.2%) codons, respectively. The codon usage is biased towards a high ratio of A/T at the third position, which is also observed in many land plant cp genomes [37].

### Sequence Comparisons with Sweet Orange

The general characteristics of the two *Citrus* cp genomes are summarized in Table 1, overall the compositions are quite similar. The GC content of these *Citrus* cp genomes is approximately 38.5%, which is slightly higher than the average of the 72 representative rosids lineages (36.7%). In these two *Citrus* cp genomes, the genic regions, introns, and intergenic regions account for ca. 58%, 11%, and 31%, respectively (Table 1).

The pairwise sequence alignment between the two *Citrus* cp genomes revealed approximately 1.3% sequence divergence (Table 2), including 1,780 indels (1.11%) and 330 substitutions (0.21%). The LSC region contains more sequence polymorphisms than expected by its size, including 1,360 (76.4%) indels and 235 (71.2%) substitutions. In contrast, the two IR regions account for ca. 34% of the cp genome yet contain only 16 (0.9%) indels and 12 (3.6%) substitutions. The size differences in the LSC and SSC regions between these two cp genomes are mostly explained by one large indel in each region. The LSC sizes differ by 596 bp and a 523-bp indel was found in the spacer between *rps16* and *trnQ-UUG*. The SSC sizes differ by 370 bp and a 354-bp indel was found in the spacer between *rpl32* and *trnL-UAA.*

**Table 2:** Differences between the *C. aurantiifolia* and *C. sinensis* cp genomes.

**Indel**				
		Length (bp)	Count	
		1	43	
		2–10	20	
		11–100	18	
		101–1,000	3	
	Sum	1,780	116	Percentage^a^: 1.11%
**Substitution**				
		Type	Count	
		A <-> T	34	
		C <-> G	15	
		A <-> C	81	
		T <-> C	64	
		A <-> G	51	
		T <-> G	85	
	Sum		330	Percentage^a^: 0.21%
**10 most divergent intergenic regions**				
		Region	Length^b^ (bp)	Pairwise distance
		*rps3* - *rpl22* (LSC)	234	0.027
		*ndhE* - *ndhG* (SSC)	276	0.018
		*psaC* - *ndhE* (SSC)	231	0.017
		*psbH* - *petB* (LSC)	118	0.017
		t*rnY-GUA-trnE-UCC* (LSC)	59	0.017
		*trnH*-*GUG* - *psbA* (LSC)	449	0.016
		*rpl32* - *trnL*-*UAG* (SSC)	1,141	0.015
		*psbT-psbN* (LSC)	66	0.015
		*trnG-GCC-trnR-UCU* (LSC)	204	0.015
		*trnD-GUC-trnY-GUA* (LSC)	469	0.013

a Relative to the length of *C. aurantiifolia.*

b Length in C. aurantiifolia.

To identify the intergenic regions that may be useful for phylogenic analysis or molecular identification, we searched for the spacers that are >400 bp in length and exhibit above-average sequence divergence between the two *Citrus* species (i.e., >1.3%). A total of three regions satisfied these criteria, including the spacer between *trnH-GUG* and *psbA* (449 bp, 1.6% divergence), the spacer between *rpl32* and *trnL-UAG* (1141 bp, 1.5% divergence), and the spacer between *trnD-GUC* and *trnY-GUA* (469 bp, 1.3% divergence).

The junctions between the IR, LSC, and SSC regions in *C*. *aurantiifolia* are similar to that of *C. sinensis* except for the LSC-IRb boundary. A total of 23 indels and five substitutions were found at this region, resulting in one copy of *rpl22* spanning across the LSC-IRb junction in *C*. *aurantiifolia*. Comparing the IR junctions of *Citrus* with *Theobroma* and *Gossypium* in Malvaceae [38], it was found that the IRs in *Citrus* have expanded to include *rps19* and 252 nt of *rpl22*, whereas in Malvaceae, *rps19* is located in LSC and *rpl22* was missing [38]–[40].

### Analyses of Repetitive Sequences

A total of 109 SSR loci were found in the cp genome of *C. aurantiifoliaa*, accounting for 1,352 bp of the total sequence (ca. 0.8%). Among these, 94 were also found in *C. sinensis* and 42 exhibit length polymorphism (Table 3). Most SSRs are located in intergenic regions, but some were found in coding genes such as *matK* and *ycf1*. Concerning the controversial status of *Citrus* taxonomy, the SSRs identified in this study may provide new perspective to refine the phylogeny and elucidate the origin of the cultivars. Furthermore, these SSRs may be used as molecular markers for population studies.

**Table 3:** List of simple sequence repeats.

Repeat unit	Length (bp)	Number of SSRs	Start position^a^
A	10	6	4512; 47812; 53871; **72614**; 121748; **159288**
	11	6	**6866**; 10130; 69481; 71892; 117725; **134802**
	12	9	**8332**; 31399; 47307; 63928; **111804**; **113977 (** ***ycf1*** **)**; 118367; 140302 (*ycf68*); 144255
	13	2	10107; 84557
	14	1	**385**
	15	1	32360
	16	2	69965; 118302
	17	3	7620; 39139; 74176
	19	1	**12023**
	22	1	70289
T	10	10	**2424 (** ***matK*** **)**; **19786**; **26964** **(** ***rpoB*** **)**; **37622**; 46938; **63632**; **87731**; 117742; 117871; 118851
	11	11	9401; 10416; **17001**; 30912; 46021; 63530; 112216; 117988; 118224; **121703**; **131189** (*ycf1*)
	12	6	**14722**; 29024; 102773; 106715 (*ycf68*); **133040 (** ***ycf1*** **)**; **135213**
	13	2	**73946**; 80423
	14	2	1776; 85274
	15	2	54209; 57817
	17	1	45965
	18	3	52748; 68339; 81409
	20	1	49202
	23	2	23694; **33282**
C	10	2	**28769**; **104247**
G	10	1	**142772**
AT	10	4	**20631 (** ***rpoC2*** **)**; **33636**; 11817; **121517 (** ***ndhD*** **)**
AAG	12	1	**97331**
AAT	12	2	**38604**; **122629**
ATA	12	1	70220
ATT	12	3	**10283**; **53810**; **54088**
	18	1	**1760**;
CTT	12	2	**37353 (** ***psbC*** **)**; **149686**
TAA	12	2	**30250**; 61945
TAT	12	1	**83297**
TTC	12	1	**73084**
TAAA	12	2	4866; **45088**
AAAT	12	3	**30423**; **32502**; **71394**
ATAC	12	1	**51167**
ATTT	12	1	49193
	20	1	**117168**
TTAA	12	2	39175; 39188
TTAG	12	1	**61483**
TTTC	12	1	**14352**
TCTT	12	1	**46961**
AATAA	20	1	144226
TTTTA	20	1	102781
TTCAAA	18	1	63817

a The SSR-containing coding regions are indicated in parentheses. SSRs that are identical in the *C. sinensis* chloroplast genome are highlighted in bold; SSRs that are conserved but with different lengths are highlighted by underline.

In addition, 62 large repeats that are longer than 30 bp were found in the *C. aurantiifolia* cp genome (Table 4). Most of these repeats are located in intergenic spacers, except for three that are located in the coding regions of *rps4*, *psaA* and *psaB.* Twelve of these long repeats were also found in *C. sinensis,* indicating that these repeats might be widespread in the genus.

**Table 4:** List of long repeat sequences.

Repeatsize	Type^a^	Start position of1st repeat	Start position the repeatfound in other region	Location^b^	Region
30	D	1759	1762	IGS (*psbA*-*trnK-UUU*)	LSC
30	P	1771	12015	IGS (*psbA*-*trnK-UUU*, *atpA*-*atpF*)	LSC
30	P	8231	37726, 47606	**IGS (** ***trnS-GCU*** **, ** ***trnS-UGA*** **, ** ***trnS-GGA*** **),**	LSC
30	D	23226	85067	**intron (** ***rpoC1*** **), IGS (** ***rpl16*** **-** ***rps3*** **)**	LSC
30	D	23686	52733	intron (*rpoC1* **)**, IGS (*ndhK*-*ndhC*)	LSC
30	P	23687	70291	intron (*rpoC1*), IGS (*trnP-UGG*-*psaJ*)	LSC
30	D	23692	33280	intron (*rpoC1*), IGS (*trnE-UUC*-*trnT-GGU*)	LSC
30	D	49192	117171	IGS (*psbA*-*trnK-UUU*), IGS (*atpA*-*atpF*)	LSC, IR
30	D, P	49197	102764, 144233	IGS (*trnT-UGU*-*trnL-UAA*), IGS (*rps12*-*trnV-GAC*)	LSC, IR
30	D, P	51215	102768, 144229	IGS (*trnF-GAA*-*ndhJ*), IGS (*rps12*-*trnV-GAC*)	LSC, IR
30	P	71344	71344	**IGS (** ***rpl33*** **-** ***rps18*** **)**	LSC
30	D, P	102768	102773, 144224	IGS (*rps12*-*trnV-GAC*)	IR
30	D	144225	144230	IGS (*trnV*-*GAC*-*rps12*)	IR
31	P	4492	117868	IGS (*trnK-UUU*-*rps16*), IGS (*rpl32*-*trnL-UAG*)	LSC, IR
31	P	10106	49188	IGS (*trnG-GCC*-*trnR-UCU*, *trnT-UGU*-*trnL-UAA*)	LSC
31	P	29811	29811	**IGS (** ***petN*** **-** ***psbM*** **)**	LSC
31	P	33281	70282	**IGS (** ***trnE-UUC*** **-** ***trnT-GGU*** **, ** ***trnP-UGG*** **-** ***psaJ*** **)**	LSC
31	P	119977	119977	intron (*ccsA*)	IR
32	D	7615	74171	IGS (*psbK*-*psbI*), intron (*clpP*)	LSC
32	P	39166	39166	IGS (*trnG-GCC*-*trnfM-CAU*)	LSC
34	P	38774	38782	**IGS (** ***psbZ*** **-** ***trnG-GCC*** **)**	LSC
34	P	49186	70288	rps4, IGS (*trnP-UGG*-*psaJ*)	LSC
34	D, P	111432	111464, 135529, 135561	**IGS (** ***rrn4.5*** **-** ***rrn5*** **)**	IR
35	P	10097	49193	IGS *trnG-GCC*-*trnR-UCU*, *trnT-UGU*-*trnL-UAA*)	LSC
36	P	27648	27648	IGS (*rpoB*-*trnC-GCA*)	LSC
40	P	77776	77776	IGS (*psbT*-*psbN*)	LSC
41	D	41294	43518	***psaB*** **, ** ***psaA***	LSC
41	D, P	102353	124945, 144633	**IGS (** ***rps12*** **-** ***trnV-GAC*** **), intron (** ***ndhA*** **)**	IR
48	P	30626	30626	**IGS (** ***petN*** **-** ***psbM*** **)**	LSC
50	D	39020	39044	**IGS (** ***trnG-GCC*** **-** ***trnfM-CAU*** **)**	LSC
51	P	9984	9984	IGS (*trnG-GCC*-*trnR-UCU*)	LSC
53	P	8869	31095	**IGS** (*trnS-GCU*-*trnG-GCC*, ***psbM*** **-** ***trnD-GUC***)	LSC
54	P	441	441	IGS (*trnH-GUG*-*psbA*)	LSC

a D: direct repeat; P: palindrome inverted repeat.

b IGS: intergenic spacer region. Sequences conserved in the *C. sinensis* chloroplast genome are highlighted in bold.

### Gene Content Analyses within the Rosids

**Figure 2:**
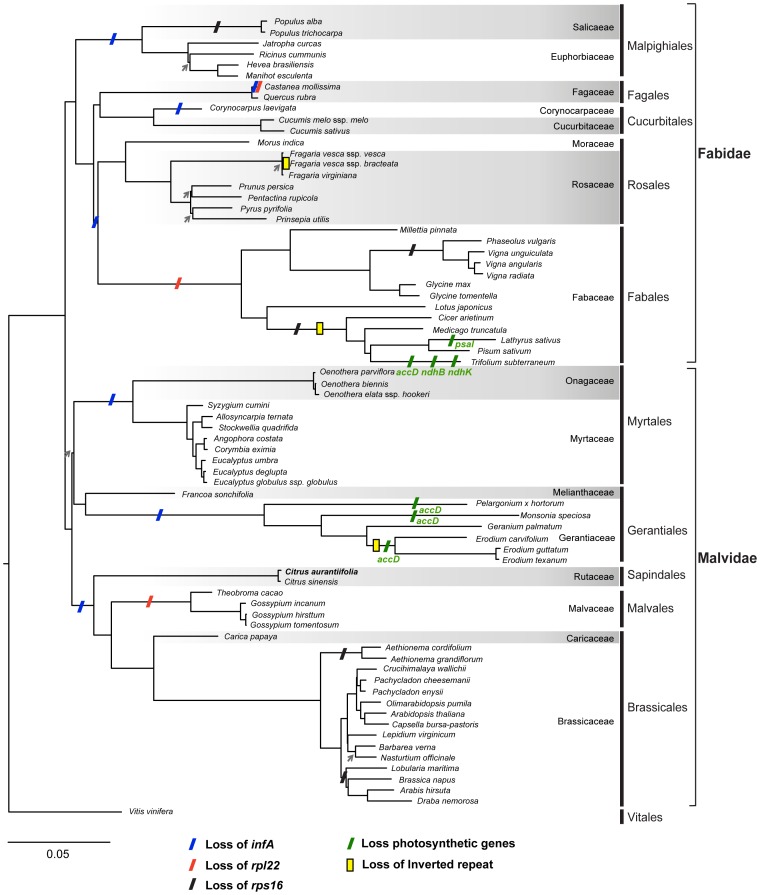
Maximum likelihood phylogeny of the representative rosids lineages. The common grape vine (*Vitis vinifera*) is included as the outgroup to root the tree. The concatenated alignment includes 62 conserved chloroplast genome genes and 54,689 aligned nucleotide sites. Nodes received <70% bootstrap support are indicated by gray arrows. The putative events of gene losses are inferred based on the most parsimonious scenario.

A maximum likelihood phylogenetic analysis of 72 representative rosids lineages was conducted based on a concatenated alignment of four rRNA and 58 protein-coding genes with 54,689 sites (Figure 2). *Citrus* represents Sapindales and is sister to the clade containing Malvales and Brassicales. These relationships are congruent with the previous reports [10], [41]–[43]. Based on this phylogeny and the gene content, we inferred the gene loss events during the cp genome evolution in rosids.

The translation initiation factor gene *infA* in cp has been lost independently at least 24 times in angiosperms and evidence provided from some cases suggested functional replacement by a nucleus copy [44]. Although the majority of *infA* in our selected cp genomes were found to be pseudogenized or completely lost, an intact *infA* was found in *Quercus*, *Francoa*, and two *Cuscumis* species.

The *rpl22* were found to be lost in Fabaceae [45] and *Castanea* of Fagaceae [46] following independent transfers to nucleus. Furthermore, another putative loss of *rpl22* was detected in *Passiflora* [46]. The *rpl22* in Malvaceae, including *Theobroma* and three *Gossypium* species, were found to be pseudogenized in our analysis. In *Citrus*, the ORF of *rpl22* was shortened to 252–264 nt compared to the typical length of 399–489 nt in other rosids [10], [46]. However, compared with the pseudogenized *rpl22* found in Malvalvace, the *rpl22* homologs in *Citrus* still show high sequence conservation. Additionally, the *rpl22* transcripts can be identified in the EST database for various *Citrus* species (data not shown). Taking account into the above consideration, we did not annotate *rpl22* as a pseudogene in *Citrus*.

The parallel losses of *rps16* were found in several rosids lineages (Figure 2), including one time in Salicaceae, two times in Fabaceae and another two times in Brassicaceae. The loss of *rps16* in *Medicago* and *Populus* was found to be substituted by a nuclear-encoded copy that transferred from the mitochondrion (mt) [47]. Because the nuclear-encoded RPS16 was found to target both mt and cp in *Arabidopsis*, *Lycopersicon*, and *Oryza* [47], it is possible that the cp genome-encoded *rps16* would not be maintained by selection and will eventually become lost in these lineages.

There are only a few gene loss events of photosynthetic genes found in rosids. In addition to the loss of *psaI* in *Lathyrus sativus* [48], the *accD* seems to be lost independently in *Trifolium subterraneum* and several Gerantiaceae species except for *Geranium palmatum*. In *Trifolium,* a nuclear-encoded *accD* copy has been reported [48], which presented another example of horizontal gene transfer from cp to nucleus. Successful gene transfers from cp to the nucleus in angiosperms are rare and have been only documented for four genes in rosids. Other than the three genes described above (i.e., *infA*, *rpl22*, and *accD*), the *rpl32* in *Populus* (Salicaceae) is the fourth example [49]–[51].

The IR has been reported to be independently lost at least five times among seed plants, two of which are within rosids [51]. In addition to the inverted repeat lacking clade (IRLC) of papilionoid Fabaceae [52] and *Erodium* of Gerantiaceae [53], [54], the IR was found to be lost in two lineages of *Fragaria* (Rosaceae), which are *F. vesca* ssp. *bracteatea* and *F. mandschurica* (accession: NC_018767, not shown in Figure 2). Based on the *Fragaria* phylogeny shown in a previous study [55], it seems that IR loss was not a single event in *Fragaria*.

### Molecular Evolution of *orf56* and *ycf68* within the Rosids

In the comparison of gene content between the two *Citrus* cp genomes, *C. aurantiifolia* was found to contain two additional protein-coding genes. The first gene, *orf56*, is located in the *trnA-UGC* intron that contains one sequence homologous to previously recognized mitochondrial *ACRS* (ACR-toxin sensitivity gene) in *Citrus* [56]. In addition to the 171-bp identical sequences between cp *orf56* and the ORF sequences of *ACRS* in mt, the full length of 355-bp region of *ACRS* that conferred sensitivity to ACR-toxin in *E. coil* are also identical. Furthermore, the whole *trnA-UGC* among two *Citrus* cp regions and *C. jambhiri* mitochondrial *ACRS* shared more than 96% identity (Figure S1), which highlight the conservation of this region between cp and mt.

**Figure 3:**
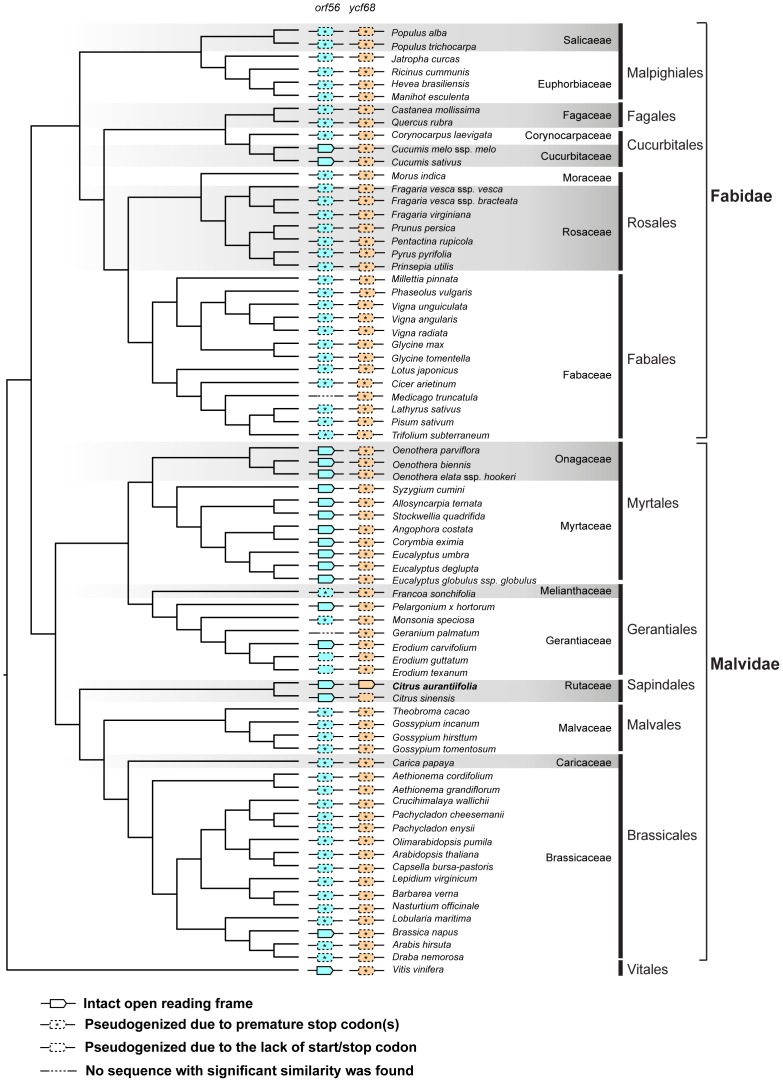
The phylogenetic distribution patterns of *orf56* and *ycf68*.

The gene *orf56* has also been included in the annotation of complete cp genomes of *Calycanthus* [57] and *Pelargonium* [58]. Our BLAST search against the rosids genome database revealed that in addition to *Citrus* and *Pelargonium*, all of the species examined in Cucurbitaceae and Myrtales also contain an intact *orf56* (Figure 3). Moreover, an intact *ACRS* ORF is also present in the mt genomes of *Liriodendron* [59] and *Silene* [60] and the ORF sequences between cp and mt are identical. Goremykin et al. [57] suggested that the *ACRS* gene was relative recently transferred from cp to mt. Based on the phylogeny containing the cp *orf56* and the mt *ACRS* (Figure S2), it appears that *orf56* has been independently transferred from cp to mt in different lineages.

The second gene, *ycf68*, is located in the *trnI-GAU* intron. A nearly identical sequence was found in *C. sinensis* but an additional T insertion near the C-terminus abolished the stop codon at the corresponding position. The intact *ycf68* can be detected in several monocots and Nymphaeaceae [61], [62]. However, in the majority of other rosids (Figure 3) and the rest of the eudicots [61], the *ycf68* homologs all contain premature stop codons. Although Raubeson et al. [61] argued that *ycf68* is not a protein-coding gene based on the lack of intron-folding pattern, the high levels of sequence conservation among the ORFs of identified homologs suggest that the true identity and functionality of this putative gene remains to be further investigated.

## Conclusions

We reported the complete cp genome sequence of *Citrus aurantiifolia* (Rutaceae) in this study. The genome organization and gene content is typical of most angiosperms and highly similar to that of *C. sinensis* (i.e., 98.7% identical at the nucleotide level). The only difference in the gene content between the two *Citrus* cp genomes is the *C. aurantiifolia*-specific presence of a protein-coding gene (*ycf68*) in the *trnI*-*GAU* intron. Notably, three long intergenic spacers with high sequence divergence and 94 shared SSR regions were identified in the *C. aurantiifolia*-*C. sinensis* comparison. These regions may provide phylogenetic utility at low taxonomic levels and could be applied to the molecular identification of *Citrus* cultivars. Finally, our comparative analysis of gene content among 72 representative rosids lineages highlighted multiple events of gene losses within this group.

## Supporting Information

Figure S1 **Alignment of the** ***orf56*** **-containing sequences of two** ***Citrus*** **cp genomes and** ***C. jambhiri*** **mitochondrial** ***ACRS*** **sequences.**
(TIF)

Figure S2 **The maximum likelihood phylogeny of the cp** ***orf56*** **and mt** ***ACRS*** **ORF sequences.**
(TIF)

Table S1 **List of the complete chloroplast genome sequences included in the phylogenetic analysis.**
(XLSX)

Table S2 **List of the genes found in the** ***C. aurantiifolia*** **cp genome.**
(XLSX)

Table S3 **Codon usage of the** ***C. aurantiifolia*** **cp genome.**
(XLSX)

## References

[1] Mabberley DJ (2004) *Citrus* (Rutaceae): a review of recent advances in etymology, systematics and medical applications. Blumea 49: 481–198.

[2] Tripoli E, Guardia ML, Giammanco S, Majo DD, Giammanco M (2007) *Citrus* flavonoids: molecular structure, biological activity and nutritional properties: a review. Food Chem 104: 466–479.

[3] Ezeabara CA, Okeke CU, Aziagba BO, Ilodibia CV, Emeka AN (2014) Determination of saponin content of various parts of six *Citrus* species. Int Res J Pure Appl Chem 4: 137–143.

[4] Nicolosi E, Deng ZN, Gentile A, La Malfa S, Continella G, et al. (2000) *Citrus* phylogeny and genetic origin of important species as investigated by molecular markers. Theor Appl Genet 100: 1155–66.

[5] Hynniewta M, Malik SK, Rao SR (2014) Genetic diversity and phylogenetic analysis of Citrus (L) from north-east India as revealed by meiosis, and molecular analysis of internal transcribed spacer region of rDNA. Meta Gene 2: 237–251. 25606407 10.1016/j.mgene.2014.01.008 PMC4287869

[6] Liu Y, Heying E, Tanumihardjo SA (2012) History, global distribution, and nutritional importance of citrus fruits. Compr Rev Food Sci Food Saf 11: 530–545.

[7] Penjor T, Yamamoto M, Uehara M, Ide M, Matsumoto N, et al (2013) Phylogenetic relationships of *Citrus* and its relatives based on *matK* gene sequences. PLoS ONE 8: e62574. 23638116 10.1371/journal.pone.0062574 PMC3636227

[8] Corriveau JL, Coleman AW (1988) Rapid screening method to detect potential biparental inheritance of plastid DNA and results for over 200 angiosperm species. Am J Bot 75: 1443–1458.

[9] Zhang Q, Liu Y (2003) Sodmergen (2003) Examination of the cytoplasmic DNA in male reproductive cells to determine the potential for cytoplasmic inheritance in 295 angiosperm species. Plant Cell Physiol 44: 941–951. 14519776 10.1093/pcp/pcg121

[10] Bausher MG, Singh ND, Lee SB, Jansen RK, Daniell H (2006) The complete chloroplast genome sequence of *Citrus sinensis* (L.) Osbeck var ‘Ridge Pineapple’: organization and phylogenetic relationships to other angiosperms. BMC Plant Biol. 6: 21. 10.1186/1471-2229-6-21 PMC1599732 17010212

[11] Davies FS, Albrigo LG (1994). Citrus. CABI International, Wiltshire, UK. 1–2.

[12] Vand SH, Abdullah TL (2012) Identification and introduction of Thornless Lime (*Citrus aurantifolia*) in Hormozgan, Iran. Indian J Sci Technol 5: 3670–3673.

[13] Al-Sadi AM, Al-Moqbali HS, Al-Yahyai RA, Al-Said FA (2012) AFLP data suggest a potential role for the low genetic diversity of acid lime (*Citrus aurantifolia* Swingle) in Oman in the outbreak of witches’ broom disease of lime. Euphytica 188: 285–297.

[14] Taheri F, Nematzadeh G, Zamharir MG, Nekouei MK, Naghavi M, et al (2011) Proteomic analysis of the Mexican lime tree response to “*Candidatus* Phytoplasma aurantifolia” infection. Mol Biosyst 7: 3028–3035. 21853195 10.1039/c1mb05268c

[15] Zamharir MG, Mardi M, Alavi SM, Hasanzadeh N, Nekouei MK, et al (2011) Identification of genes differentially expressed during interaction of Mexican lime tree infected with “*Candidatus* Phytoplasma aurantifolia”. BMC Microbiol. 11: 1. 10.1186/1471-2180-11-1 PMC3271359 21194490

[16] Monavarfeshani A, Mirzaei M, Sarhadi E, Amirkhani A, Khayam Nekouei M, et al (2013) Shotgun proteomic analysis of the Mexican lime tree infected with “*Candidatus* Phytoplasma aurantifolia.”. J Proteome Res 12: 785–795. 23244174 10.1021/pr300865t

[17] Faghihi MM, Bagheri AN, Bahrami HR, Hasanzadeh H, Rezazadeh R, et al (2011) Witches’-broom disease of lime affects seed germination and seedling growth but is not seed transmissible. Plant Disease 95: 419–422. 10.1094/PDIS-06-10-0400 30743329

[18] Zafari S, Niknam V, Musetti R, Noorbakhsh SN (2012) Effect of phytoplasma infection on metabolite content and antioxidant enzyme activity in lime (*Citrus aurantifolia*). Acta Physiol Plant 34: 561–568.

[19] Maixner M, Ahrens U, Seemüller E (1995) Detection of the German grapevine yellows (Vergilbungskrankheit) MLO in grapevine, alternative hosts and a vector by a specific PCR procedure. Eur J Plant Pathol 101: 241–250.

[20] Ku C, Hu J-M, Kuo C-H (2013) Complete plastid genome sequence of the basal asterid *Ardisia polysticta* Miq. and comparative analyses of asterid plastid genomes. PLoS ONE 8: e62548. 23638113 10.1371/journal.pone.0062548 PMC3640096

[21] Ku C, Chung W-C, Chen L-L, Kuo C-H (2013) The complete plastid genome sequence of Madagascar periwinkle *Catharanthus roseus* (L.) G. Don: plastid genome evolution, molecular marker identification, and phylogenetic implications in asterids. PLoS ONE 8: e68518. 23825699 10.1371/journal.pone.0068518 PMC3688999

[22] Zerbino DR, Birney E (2008) Velvet: algorithms for *de novo* short read assembly using de Bruijn graphs. Genome Res 18: 821–829. 18349386 10.1101/gr.074492.107 PMC2336801

[23] Li H, Durbin R (2009) Fast and accurate short read alignment with Burrows-Wheeler transform. Bioinformatics. 25: 1754–1760. 10.1093/bioinformatics/btp324 PMC2705234 19451168

[24] Li H, Handsaker B, Wysoker A, Fennell T, Ruan J, et al (2009) The Sequence Alignment/Map format and SAMtools. Bioinformatics 25: 2078–2079. 19505943 10.1093/bioinformatics/btp352 PMC2723002

[25] Robinson JT, Thorvaldsdottir H, Winckler W, Guttman M, Lander ES, et al (2011) Integrative genomics viewer. Nat Biotechnol 29: 24–26. 21221095 10.1038/nbt.1754 PMC3346182

[26] Wyman SK, Jansen RK, Boore JL (2004) Automatic annotation of organellar genomes with DOGMA. Bioinformatics 20: 3252–3255. 15180927 10.1093/bioinformatics/bth352

[27] Altschul SF, Gish W, Miller W, Myers EW, Lipman DJ (1990) Basic local alignment search tool. J Mol Biol 215: 403–410. 2231712 10.1016/S0022-2836(05)80360-2

[28] Camacho C, Coulouris G, Avagyan V, Ma N, Papadopoulos J, et al (2009) BLAST+: architecture and applications. BMC Bioinformatics 10: 421. 20003500 10.1186/1471-2105-10-421 PMC2803857

[29] Charif D, Lobry JR (2007) SeqinR 1.0–2: a contributed package to the R project for statistical computing devoted to biological sequences retrieval and analysis. In: Bastolla DU, Porto PDM, Roman DHE, Vendruscolo DM, editors. Structural Approaches to Sequence Evolution. Biological and Medical Physics, Biomedical Engineering. Springer Berlin Heidelberg. 207–232.

[30] Lohse M, Drechsel O, Bock R (2007) OrganellarGenomeDRAW (OGDRAW): a tool for the easy generation of high-quality custom graphical maps of plastid and mitochondrial genomes. Curr Genet 52: 267–274. 17957369 10.1007/s00294-007-0161-y

[31] Darling ACE, Mau B, Blattner FR, Perna NT (2004) Mauve: multiple alignment of conserved genomic sequence with rearrangements. Genome Res 14: 1394–1403. 15231754 10.1101/gr.2289704 PMC442156

[32] Edgar RC (2004) MUSCLE: multiple sequence alignment with high accuracy and high throughput. Nucl Acids Res 32: 1792–1797. 15034147 10.1093/nar/gkh340 PMC390337

[33] Felsenstein J (1989) PHYLIP - Phylogeny Inference Package (version 3.2). Cladistics 5: 164–166.

[34] Kurtz S, Schleiermacher C (1999) REPuter: fast computation of maximal repeats in complete genomes. Bioinformatics 15: 426–427. 10366664 10.1093/bioinformatics/15.5.426

[35] Guindon S, Gascuel O (2003) A simple, fast, and accurate algorithm to estimate large phylogenies by maximum likelihood. Syst Biol 52: 696–704. 14530136 10.1080/10635150390235520

[36] Li L, Stoeckert CJ, Roos DS (2003) OrthoMCL: Identification of ortholog groups for eukaryotic genomes. Genome Res 13: 2178–2189. 12952885 10.1101/gr.1224503 PMC403725

[37] Clegg MT, Gaut BS, Learn GH, Morton BR (1994) Rates and patterns of chloroplast DNA evolution. Proc Natl Acad Sci U S A 91: 6795–6801. 8041699 10.1073/pnas.91.15.6795 PMC44285

[38] Kane N, Sveinsson S, Dempewolf H, Yang JY, Zhang D, et al (2012) Ultra-barcoding in cacao (*Theobroma* spp. Malvaceae) using whole chloroplast genomes and nuclear ribosomal DNA. Am J Bot 99: 320–329. 22301895 10.3732/ajb.1100570

[39] Lee SB, Kaittanis C, Jansen RK, Hostetler JB, Tallon LJ, et al (2006) The complete chloroplast genome sequence of *Gossypium hirsutum*: organization and phylogenetic relationships to other angiosperms. BMC Genomics 7: 61. 16553962 10.1186/1471-2164-7-61 PMC1513215

[40] Xu Q, Xiong G, Li P, He F, Huang Y, et al (2012) Analysis of complete nucleotide sequences of 12 *Gossypium* chloroplast genomes: origin and evolution of allotetraploids. PLoS ONE 7: e37128. 22876273 10.1371/journal.pone.0037128 PMC3411646

[41] Bremer B, Bremer K, Chase MW, Fay MF, Reveal JL, et al (2009) An update of the Angiosperm Phylogeny Group classification for the orders and families of flowering plants: APG III. Bot J Linn Soc 161: 105–121.

[42] Worberg A, Alford MH, Quandt D, Borsch T (2009) Huerteales sister to Brassicales plus Malvales, and newly circumscribed to include *Dipentodon*, *Gerrardina*, *Huertea*, *Perrottetia*, and *Tapiscia* . Taxon 58: 468–478.

[43] Ruhfel BR, Gitzendanner MA, Soltis PS, Soltis DE, Burleigh JG (2014) From algae to angiosperms-inferring the phylogeny of green plants (*Viridiplantae*) from 360 plastid genomes. BMC Evol Biol. 14: 23. 10.1186/1471-2148-14-23 PMC3933183 24533922

[44] Millen RS, Olmstead RG, Adams KL, Palmer JD, Lao NT, et al (2001) Many parallel losses of *infA* from chloroplast DNA during angiosperm evolution with multiple independent transfers to the nucleus. Plant Cell 13: 645–658. 11251102 10.1105/tpc.13.3.645 PMC135507

[45] Gantt JS, Baldauf SL, Calie PJ, Weeden NF, Palmer JD (1991) Transfer of *rpl22* to the nucleus greatly preceded its loss from the chloroplast and involved the gain of an intron. EMBO J 10: 3073–3078. 1915281 10.1002/j.1460-2075.1991.tb07859.x PMC453023

[46] Jansen RK, Saski C, Lee S-B, Hansen AK, Daniell H (2011) Complete plastid genome sequences of three rosids (*Castanea*, *Prunus*, *Theobroma*): evidence for at least two independent transfers of *rpl22* to the nucleus. Mol Biol Evol 28: 835–847. 20935065 10.1093/molbev/msq261 PMC3108605

[47] Ueda M, Nishikawa T, Fujimoto M, Takanashi H, Arimura S, et al (2008) Substitution of the gene for chloroplast RPS16 was assisted by generation of a dual targeting signal. Mol Biol Evol 25: 1566–1575. 18453549 10.1093/molbev/msn102

[48] Magee AM, Aspinall S, Rice DW, Cusack BP, Sémon M, et al (2010) Localized hypermutation and associated gene losses in legume chloroplast genomes. Genome Res 20: 1700–1710. 20978141 10.1101/gr.111955.110 PMC2989996

[49] Cusack BP, Wolfe KH (2007) When gene marriages don’t work: divorce by subfunctionalization. Trends Genet 23: 270–272. 17418444 10.1016/j.tig.2007.03.010

[50] Ueda M, Fujimoto M, Arimura S, Murata J, Tsutsumi N, et al (2007) Loss of the *rpl32* gene from the chloroplast genome and subsequent acquisition of a preexisting transit peptide within the nuclear gene in *Populus* . Gene 402: 51–56. 17728076 10.1016/j.gene.2007.07.019

[51] Jansen RK, Ruhlman TA (2012) Plastid genomes of seed plants. In: Bock R, Knoop V, editors. Genomics of chloroplasts and mitochondria. Advances in photosynthesis and respiration. Springer Netherlands. 103–126.

[52] Wojciechowski MF, Lavin M, Sanderson MJ (2004) A phylogeny of legumes (Leguminosae) based on analysis of the plastid *matK* gene resolves many well-supported subclades within the family. Am J Bot 91: 1846–1862. 21652332 10.3732/ajb.91.11.1846

[53] Blazier JC, Guisinger MM, Jansen RK (2011) Recent loss of plastid-encoded *ndh* genes within *Erodium* (Geraniaceae). Plant Mol Biol 76: 263–272. 21327834 10.1007/s11103-011-9753-5

[54] Guisinger MM, Kuehl JV, Boore JL, Jansen RK (2011) Extreme reconfiguration of plastid genomes in the angiosperm family Geraniaceae: rearrangements, repeats, and codon usage. Mol Biol Evol 28: 583–600. 20805190 10.1093/molbev/msq229

[55] Njuguna W, Liston A, Cronn R, Ashman TL, Bassil N (2013) Insights into phylogeny, sex function and age of *Fragaria* based on whole chloroplast genome sequencing. Mol Phylogenet Evol 66: 17–29. 22982444 10.1016/j.ympev.2012.08.026

[56] Ohtani K, Yamamoto H, Akimitsu K (2002) Sensitivity to *Alternaria alternata* toxin in citrus because of altered mitochondrial RNA processing. Proc Natl Acad Sci U S A 99: 2439–2444. 11842194 10.1073/pnas.042448499 PMC122383

[57] Goremykin V, Hirsch-Ernst KI, Wölfl S, Hellwig FH (2003) The chloroplast genome of the “basal” angiosperm *Calycanthus fertilis* – structural and phylogenetic analyses. Plant Syst Evol 242: 119–135.

[58] Chumley TW, Palmer JD, Mower JP, Fourcade HM, Calie PJ, et al (2006) The complete chloroplast genome sequence of *Pelargonium* × *hortorum*: organization and evolution of the largest and most highly rearranged chloroplast genome of land plants. Mol Biol Evol 23: 2175–2190. 16916942 10.1093/molbev/msl089

[59] Richardson AO, Rice DW, Young GJ, Alverson AJ, Palmer JD (2013) The “fossilized” mitochondrial genome of *Liriodendron tulipifera*: ancestral gene content and order, ancestral editing sites, and extraordinarily low mutation rate. BMC Biol 11: 29. 23587068 10.1186/1741-7007-11-29 PMC3646698

[60] Sloan DB, Müller K, McCauley DE, Taylor DR, Šorchová H (2012) Intraspecific variation in mitochondrial genome sequence, structure, and gene content in *Silene vulgaris*, an angiosperm with pervasive cytoplasmic male sterility. New Phytol 196: 1228–1239. 23009072 10.1111/j.1469-8137.2012.04340.x

[61] Raubeson LA, Peery R, Chumley TW, Dziubek C, Fourcade HM, et al (2007) Comparative chloroplast genomics: analyses including new sequences from the angiosperms *Nuphar advena* and *Ranunculus macranthus* . BMC Genomics 8: 174. 17573971 10.1186/1471-2164-8-174 PMC1925096

[62] Ahmed I, Biggs PJ, Matthews PJ, Collins LJ, Hendy MD, et al (2012) Mutational dynamics of aroid chloroplast genomes. Genome Biol Evol 4: 1316–1323. 23204304 10.1093/gbe/evs110 PMC3542561

