# Supplementary figures and images for: Complete Chloroplast Genome Sequence of Omani Lime (Citrus aurantiifolia) and Comparative Analysis within the Rosids
Source: PLoS One. 2014 Nov 14;9(11):e113049. doi: 10.1371/journal.pone.0113049 (PMC4232571; doi:10.1371/journal.pone.0113049)

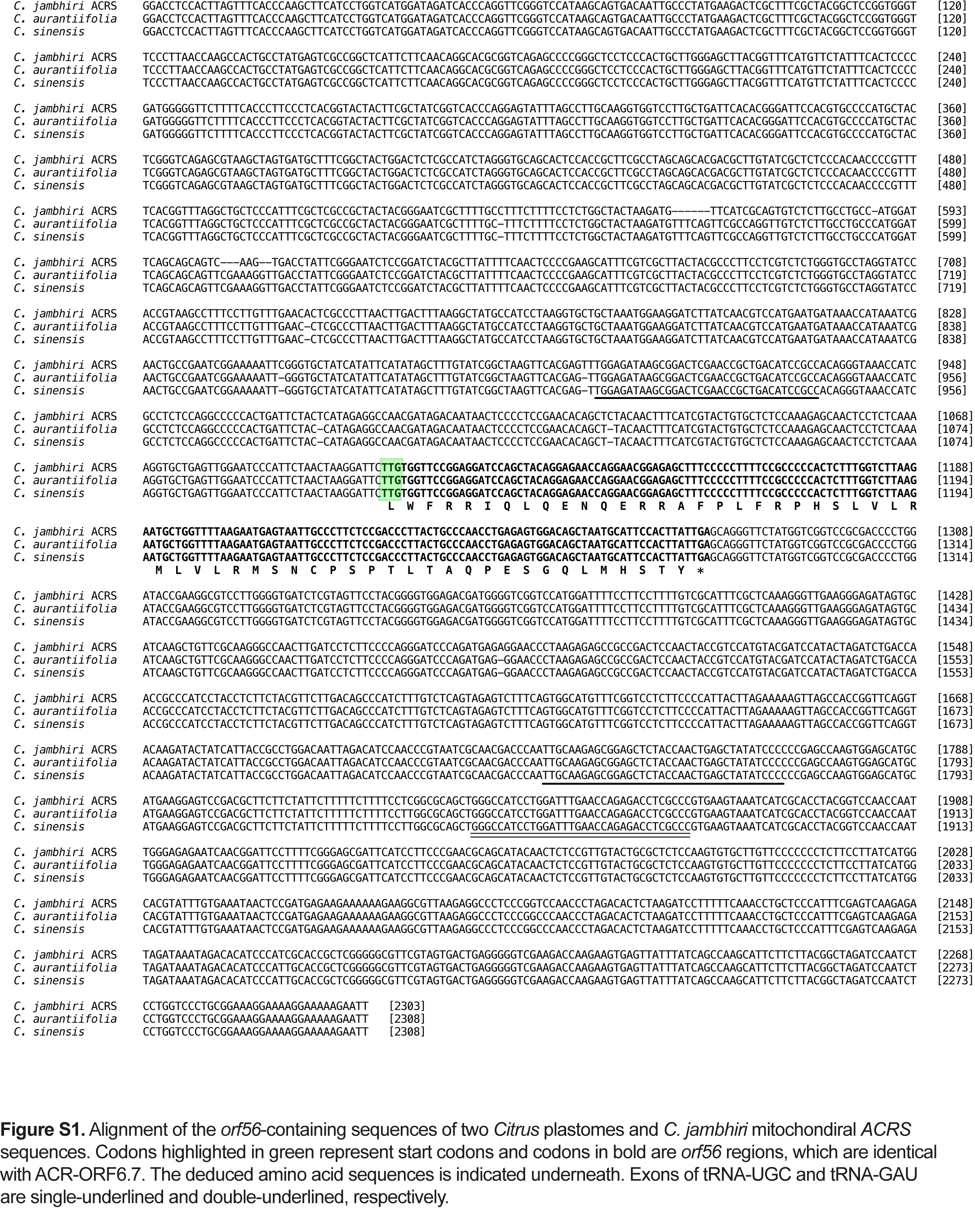

Supplement: Figure S1 — Alignment of the orf56 -containing sequences of two Citrus cp genomes and C. jambhiri mitochondrial ACRS sequences. (TIF) [file pone.0113049.s001.tif]

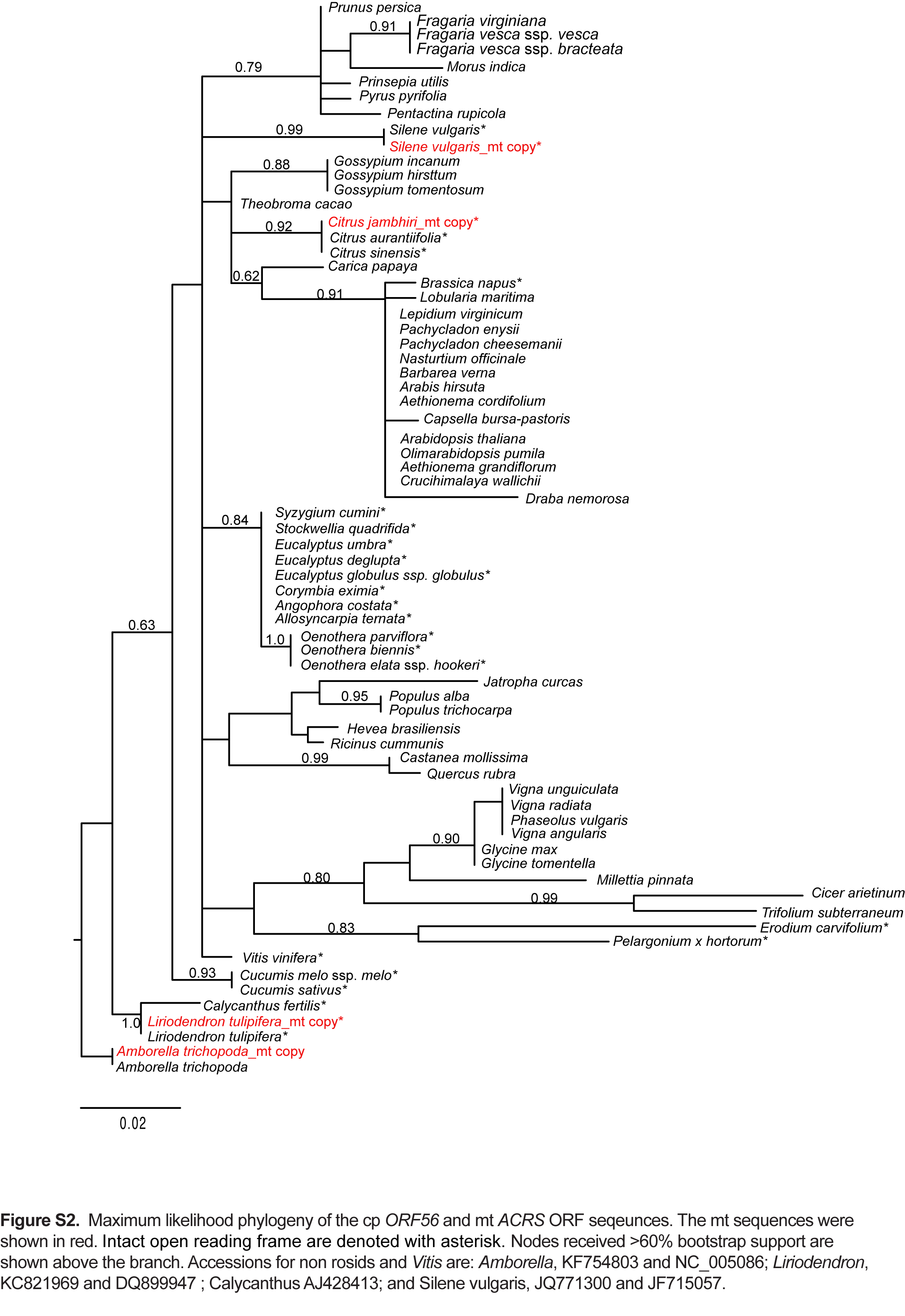

Supplement: Figure S2 — The maximum likelihood phylogeny of the cp orf56 and mt ACRS ORF sequences. (TIF) [file pone.0113049.s002.tif]
